# Neuropeptide Y modulates fear and fear extinction in distinct nuclei of the amygdala

**DOI:** 10.1186/2050-6511-13-S1-A87

**Published:** 2012-09-17

**Authors:** Ramon O Tasan, Dilip Verma, Mario Mietzsch, Regine Heilbronn, Herbert Herzog, Günther Sperk

**Affiliations:** 1 Institute of Pharmacology, Innsbruck Medical University, 6020 Innsbruck, Austria; 2 Institute of Virology, Charité, Campus Benjamin Franklin, Free University of Berlin, 12203 Berlin, Germany; 3 Neuroscience Research Program, Garvan Institute of Medical Research, Darlinghurst NSW 2010, Australia

## Background

Fear and anxiety are integrated in the amygdaloid nuclei and involve the interplay of the amygdala with various other brain areas. Neuropeptides play a critical role in regulating these processes. Neuropeptide Y (NPY) is highly expressed in limbic brain areas, including the amygdala. Depending on the receptor subtypes involved (Y_1_, Y_2_ or Y_4_), NPY has different, in part opposing effects on anxiety, fear and depression-related behaviors.

## Methods

We combined site-specific deletion of NPY receptors and locally restricted over-expression of NPY receptor subtype-selective ligands with behavioral analysis to elucidate the contribution of the individual receptor subtypes in the modulation of emotional behavior.

## Results

In Pavlovian fear conditioning, NPY knock-out (KO) mice display a dramatically accelerated acquisition of conditioned fear while fear extinction was impaired. Interestingly this phenotpye was only reproduced in mice lacking both the Y_1_ and the Y_2_ receptor. In Y_1_ single KO mice acquisition was moderately faster while fear extinction was delayed. Deletion of NPY and in particular of Y_2_ receptors resulted also in a generalization of cued as well as context fear. Local over-expression of NPY by an rAAV vector in the basolateral amygdala delayed the acquisition and facilitated the extinction of fear, both in WT and NPY KO mice, emphasizing the crucial role of this area in NPY-mediated fear acquisition and extinction. On the other hand, deletion of Y_2_ receptors in the central amygdala resulted in an increased expression and delayed extinction of conditioned fear, while there was no change in fear acquisition.

## Conclusions

Taken together, our data demonstrate that NPY delays acquisition and reduces expression of conditioned fear whereas it promotes fear extinction. Both Y_1_ and Y_2_ receptors are involved in these processes. Y_1_ receptors in the basolateral amygdala are modulating the acquisition and extinction of fear while Y_2_ receptors in the central amygdala are preferentially inhibiting the expression but facilitating the extinction of learned fear. Furthermore, Y_2_ receptors are crucially involved in the discrimination of fear-related stimuli.

